# Cross-cultural adaptation and validation of the Italian Spine Youth Quality of Life (ISYQOL) questionnaire's Arabic version

**DOI:** 10.1097/MD.0000000000028063

**Published:** 2021-12-10

**Authors:** Salah M. Fallatah, Shaker Emam, Ghamid Al-Ghamdi, Faisal Almatrafi

**Affiliations:** a Department of Orthopedics, Faculty of Medicine, Umm Al-Qura University, Makkah, KSA; b Faculty of Medicine, Umm Al-Qura University, Makkah, KSA.

**Keywords:** cross-cultural adaptation, health-related quality of life, ISYQOL, questionnaires, scoliosis, spinal deformity, SRS-22r

## Abstract

Health-related quality of life (HRQOL) assessment is considered among the most relevant outcome measures following conservative and surgical interventions for various spinal conditions. Several questions are available to evaluate HRQOL in these conditions. A more recent Italian Spine Youth Quality of Life (ISYQOL) questionnaire was developed for this purpose and showed high validity in measuring HRQOL.

To translate and adapt the ISYQOL questionnaire into Arabic language (ISYQOL-Ar) and evaluate its validity and reliability, and to correlate it with the validated revised Scoliosis Research Society (SRS-22r)'s Arabic questionnaire in a cross-sectional multicenter study.

The ISYQOL was translated, back-translated, and reviewed by an expert committee. Reliability assessment for the questionnaire domains was performed using Cronbach's alpha. For construct validation, the Pearson's correlation coefficient was used.

A total of 115 patients were enrolled in the study and completed the ISYQOL-Ar and Arabic SRS-22r questionnaires. A total of 72 patients (63%) completed the first set of questionnaires, and 2 weeks later, 63 patients (55%) completed both sets of questionnaires, with 15.8 a mean age, 39.5° mean Cobb angle of 88.9% females. ISYQOL-Ar showed excellent validity, good reliability, and internal consistency for spine health and brace wear, with Cronbach's alpha > 0.6, similar to SRS-22r in the same cohort. The correlation was significant between ISYQOL-Ar and Arabic SRS-22r (Pearson's coefficient = 0.708, *P* < .001).

The ISYQOL-Ar questionnaire is a reliable and valid outcome measure for the assessment of young patients with spinal deformity among the Arabic-speaking population.

## 1 Introduction

Patients with spinal deformities such as Scheuermann juvenile kyphosis and adolescent idiopathic scoliosis, might be influenced by their everyday activities and overall quality of life due to the psychosocial and physical symptoms associated with their conditions, regardless of the degree of their spinal deformity, with a significant effect on the health-related quality of life (HRQOL).^[1–4]^ Therefore, HRQOL assessment is highly recommended.^[5–9]^ It reflects the good quality of care, which helps evaluate the effectiveness of the medical intervention, whether it is observation, bracing, or surgery, in addition to the standard radiographic evaluation. Furthermore, assessing patients’ quality of life can help physicians manage their patients.^[10–15]^

Several questionnaires have been developed in the past to assess HRQOL in patients with spinal deformities. The standard and most used tool to assess HRQOL in patients with scoliosis is the Scoliosis Research Society 22 (SRS-22r) questionnaire.^[16]^ It comprises 5 domains: function, self-image, pain, mental health, and satisfaction with management; the first 4 domains consist of 5 questions, where the last domain consists of 2 questions only.^[17]^ In recent years, it has undergone several adaptations and validations into multiple languages worldwide, including the Arabic language.^[13,14,17–28]^ The Italian Spine Youth Quality of Life (ISYQOL) questionnaire is a new evaluation tool that was developed using Rasch analysis. It is intended to measure the quality of life of young patients with spinal deformities. This was evaluated using Rasch analysis and showed good measurement properties. Rasch analysis is a valuable and practical statistical model designed to improve the development of valid and high-quality questionnaires. It has several advantages over the classic test theory, which is used in the development of SRS-22r.^[12,15,29]^ Moreover, the ISYQOL questionnaire showed higher validity in assessing HRQOL than the SRS-22r using Spearman's correlation coefficient with better detection of the difference between groups related to curve type, brace wear, trunk appearance, and age.^[30]^

In the field of medicine, global information exchange is required to improve health care worldwide, as people benefit from others’ experiences. Cross-cultural studies have proven their importance in helping physicians to evaluate patients, especially in quality-of-life assessments.^[5,31,32]^ Internationally, questionnaires in different medical fields have been translated and validated in different languages.^[31]^ The ISYQOL questionnaire is considered new in the field, and its applications in patients from different cultures, ethnic backgrounds, and countries is still needed. Its validation in different languages, including Arabic, is still lacking. This study aims to translate, adapt, and validate a new Arabic version of the ISYQOL questionnaire (ISYQOL-Ar). Validity, reliability, and internal consistency were evaluated and correlated with the validated revised SRS-22r Arabic version.

## 2 Materials and methods

### 2.1 Study design

This is a comparative cross-sectional study to adapt and validate the Arabic version of the ISYQOL Arabic version. We addressed 3 main components: translation, reliability assessment, and validation, between the SRS-22r and the new ISYQOL questionnaire. This study was translated according to the recommendations and guidelines outlined by Tsang et al.^[33]^ Before conducting the study, ethical approval was obtained from the Committee of Biomedical Ethics of the Faculty of Medicine of Umm Al-Qura University.

### 2.2 Forward translation

The ISYQOL English version was translated to Arabic by 2 independent bilingual translators, with the Arabic language being their mother tongue. The first translator was aware of the objectives and concepts of the study, while the second translator was unaware of them, with each one performing the task independent of the other. This was performed to detect subtle differences and errors in the first translation. After reviewing both forward translations, disagreements and discrepancies were resolved through discussion. A reconciliation process was performed to develop a single forward translated Arabic version of the ISYQOL questionnaire.^[34]^

### 2.3 Backward translation

The forward Arabic translated version of the ISYQOL questionnaire was independently translated back into English. This was performed by 2 independent translators who used English as their mother tongue. Both were unaware of the intended concepts of the survey items and were blinded to the original English version of the ISYQOL questionnaire. After reviewing both backward translations, reconciliation was performed to develop a single backward translation with discrepancies and disagreements resolved by discussion in the forward translation step.

### 2.4 Expert committee assessment and content validation

All translated versions, in addition to the original ISYQOL questionnaire, were submitted to a panel of experts for review. The expert committee, which consisted of a spine surgery professor, translators, back-translators, a professional translator, and a recording observer), ensured that the final translation and original version had achieved idiomatic, semantic, conceptual, and experimental equivalence. All other elements of the questionnaire, such as instructions and section titles, were evaluated. After documenting all the steps in a written report, the expert committee formed a prefinal version of the ISYQOL-Ar questionnaire.

### 2.5 Preliminary pilot testing

**Figure 1:**
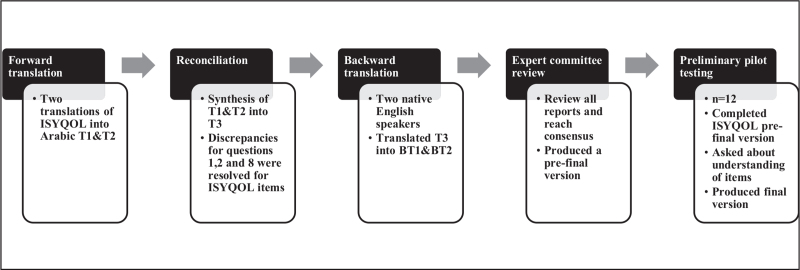
Cross-cultural adaptation stages. (ISYQOL = Italian Spine Youth quality of life questionnaire, T1 = translation 1, T2 = translation 2, T3 = translation 3, BT1 = back word translation 1, BT2 = back word Translation 2, n = number).

Twelve consecutive Arabic-speaking patients with adolescent idiopathic scoliosis (age 11–18 years) completed the prefinal version of the ISYQOL-Ar questionnaire. They were separately asked about their perception of the items, answers, and the length of time taken to complete the questionnaire. Based on the patient's responses, the expert committee developed a vinal version to be tested. These 12 patients were included in the final analysis but were too small to be analyzed separately (Fig. 1).

### 2.6 Reliability assessment

The reliability assessment depends on internal consistency, which reflects the extent to which a questionnaire item is intercorrelated. Internal consistency was measured using Cronbach's alpha coefficients for each questionnaire domain. A higher coefficient value reflects higher reliability. Values were interpreted as unacceptable for less than 0.5, poor for 0.5 to 0.59, acceptable for 0.6 to 0.69, good for 0.7 to 0.89, and excellent for 0.9 or more.^[17]^

### 2.7 Construct validation and concurrent validity

Construct validity evaluates whether a test measures what it is intended to measure. The same group of patients completed both Arabic versions of the SRS-22r and ISYQOL questionnaires. The measurements of construct validation depended on the correlation between the 2 expected patterns that may be achieved from the questionnaires. We expected that the total scores of the 2 questionnaires would correlate well, as both were supposed to measure the same construction, which is the quality of life. The domains of both questionnaires were correlated. The correlation was determined using the Pearson's coefficient. Correlation values were considered poor for less than 0.2, low for 0.2to 0.49, good for 0.5to 0.7, and strong for more than 0.7.^[17]^

### 2.8 Patient population and statistical analysis

This study was conducted over a 4-month period in 3 outpatient spine clinics in the western region of Saudi Arabia, where 115 Arabic-speaking patients known to have adolescent idiopathic scoliosis or Scheuermann kyphosis were contacted to participate in the study. Patients with neurological, congenital, or chronic diseases were excluded from the study. In addition, only patients aged 11 to 18 years at the time of diagnosis were included. After obtaining consent from the patients, they were given the final versions of the ISYQOL-Ar and the validated Arabic version of the SRS-22r questionnaire to fill and return within 4 weeks. Two weeks after receiving their response or after 4 weeks from sending the first set of questionnaires, a second final Arabic version of the ISYQOL questionnaire was sent to all participants with a request to have the questionnaire filled and returned to evaluate the test–retest reliability. The test–retest reliability of the ISYQOL-Ar was measured using a correlation coefficient with a 95% confidence interval.^[17]^ The SPSS 27.0 for Windows (SPSS Inc, Chicago, IL) was used for the statistical analysis.

## 3 Results

### 3.1 Patient sample

No changes or adjustments to the questionnaire were required after the prefinal testing. All 12 patients understood, answered, and completed the ISYQOL-Ar questionnaire within 7 minutes without any difficulties. Of the 115 patients who were contacted to participate in the study, 72 patients (62%) fulfilled the questionnaires’ first set, and 63 patients (55%) fulfilled the questionnaires’ both sets with 21.3 (± 9.2) a response time of questionnaires. The mean age of the patients was 15.8 (range 11–18 years), 88.8% were female, and the curve magnitude ranged between 15° and 80° (mean 39.5 ± 18). Thirty-nine of the patients had type I and II thoracic curves according to Lenke classification, and 33 had other curve types (Lenke III, IV, V, or VI).^[35]^ Of the 72 patients, 25 were under observation, 13 were treated with a brace application, and 34 patients were managed by surgical scoliosis correction with no notable differences between the 3 groups in terms of sex or curve type. The basic characteristics and demographics of all the participants are summarized in Table 1.

**Table 1:** Characteristics of the study participants.

Age	
Mean age (yr)	15.8 (10–18)
Gender	
Male (%)	8 (11.1%)
Female (%)	64 (88.8%)
Disease management	
Observation	25
Brace	13
Surgery	34

### 3.2 Content analysis

The ISYQOL questionnaire consisted of 2 domains: the spine health domain composed of 13 questions and the brace domain composed of 7 questions. The ISYQOL questions were scored from 0 (best score) to 2 (worst score). Grey questions (questions 5, 6, 10, and 13) scored 0 for often, 1 for sometimes, and 2 for never. Conversely, the remaining questions are called white questions and scored 0 for never, 1 for sometimes, and 2 for often. Based on the Rasch-consistent quality of life measurement, the lower the result, the better the quality of life. Prior to analyzing the data, the ISYQOL items were reverse-coded, and the final score was expressed as a percentage of 100%, suggesting the best HRQOL. The SRS-22r consists of 5 domains: function, pain, mental health, self-image, and satisfaction with management. Every domain was composed of 5 questions, except for satisfaction with only 2 questions. Every question had 5 answers that were scored from 5, suggesting a maximum score of 1, suggesting the minimum. In the SRS-22r scoring, patients with higher results correlated with better HRQOL.

In our study, the mean percentage of the final version of the ISYQOL-Ar questionnaire was 74 (± 12.1), whereas the Arabic SRS-22r main domain scores were between 3.9 and 4.3. Neither ceiling nor floor effects were observed with the ISYQOL-Ar questionnaire. In contrast, a ceiling effect was observed for pain, self-image, mental health, and satisfaction with management domains with the Arabic SRS-22r (Table 2). Both the ISYQOL-Ar and Arabic SRS-22 questionnaires showed that mild curves, having a good trunk appearance, and not wearing a brace correlated with a significantly better quality of life.

**Table 2:** Descriptive statistics on individual domain scores.

Questionnaire/domain (no. of questions)	Mean (SD)	Minimum	Maximum	% Floor	% Ceiling
ISYQOL-Ar^∗^					
Spine health (13)	0.67 (0.35)	0.15	1.85	0	0
Brace (7)	0.84 (0.38)	0.43	1.71	0	0
SRS-22r^†^					
Pain (5)	3.95 (0.66)	2.4	5	0	5.9
Function (5)	3.91 (0.61)	1.6	4.6	0	0
Self-image (5)	4.09 (0.74)	1.4	5	0	5.9
Mental health (5)	3.99 (0.73)	2.4	5	0	5.9
Satisfaction with management (2)	4.39 (0.83)	1.5	5	0	41.2

### 3.3 Reliability

The reproducibility of spine health and brace wear assessment, both measured by test–retest reliability for the ISYQOL-Ar, showed promising results with a Cronbach's alpha of 0.798 for spine health and 0.554 for brace wear. The different domains of the Arabic SRS-22r measured from 0.665 to 0.788, indicating good internal consistency and test–retest reliability (Table 3).

**Table 3:** Internal consistency and reliability assessment.

ISYQOL-Ar	Cronbach's α	SRS-22r	Cronbach's α
Spine health	0.798	Pain	0.675
brace	0.554	Function	0.665
		Mental health	0.788
		Self-image	0.778
		Satisfaction with management	0.714

### 3.4 Validity

**Figure 2:**
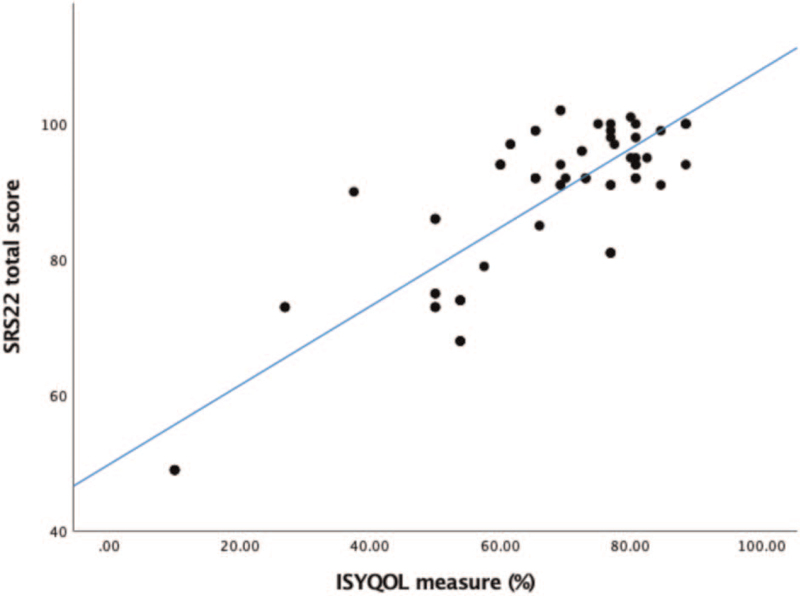
Correlation between ISYQOL and SRS22r scores showing excellent validity using Pearson's correlation (Pearson's coefficient = 0.708; *P* value < .001). ISYQOL = Italian Spine Youth Quality of life questionnaire, SRS22r = revised version of Scoliosis research Society 22 Questionnaire.

The correlation between the ISYQOL-Ar and Arabic SRS-22r was determined using Pearson's correlation coefficient. An overall significantly strong correlation was found between the 2 questionnaires, with a Pearson's coefficient of 0.708 (*P* < .001) (Fig. 2). Further analysis of the correlation between different domains of the 2 questionnaires showed significant correlations except for the Brace domain of ISYQOL-Ar and the pain and function domains of SRS-22r, which might be attributed to the small number of patients wearing the brace in this study group (Table 4).

**Table 4:** Validity using Pearson's coefficient between ISYQOL-Ar and SRS-22r questionnaires.

ISYQOL-Ar	SRS-22r				
	Pain	Function	Mental health	Self-image	Satisfaction with management
Spine health	0.706^∗^	0.481^∗^	0.520^∗^	0.628^∗^	0.381^∗^
Brace	0.580	0.393	0.725^†^	0.889^∗^	0.751^†^

## 4 Discussion

Using international guidelines, this study demonstrates the cross-cultural adaptation and translation of the English version of the ISYQOL questionnaire into the Arabic language, testing its psychometric properties, test–retest reliability, internal consistency, and construct validity. The ISYQOL questionnaire is a Rasch-consistent tool used to assess HRQOL in adolescents with spinal deformities. It is considered relatively new in the field compared with other well-validated and translated questionnaires. SRS-22r is one of the accepted and most used tools for measuring HRQOL in scoliosis and other spinal deformities that have been previously adapted for different languages and cultures.^[12,15,30]^ Such studies are very important to avoid vague instructions, items, or statements specific to certain cultures. This condition may provoke different manifestations in different cultures and ethnicities, which may be attributed to lifestyle and other local factors.^[36]^

In our study, we translated, validated, and culturally adapted the English version of the ISYQOL into the Arabic language. The authors ensured equivalence between both languages for the ISYQOL questionnaire by strictly adhering to the international guidelines on the topic. In each step of the translation process, the investigators thoroughly read, reviewed, discussed, cross-checked, and proofread all translated versions and reached a consensus on all discrepancies. All patients described the questionnaire as simple, straightforward, understandable, and easy to complete.

In this study, all translated versions have been thoroughly discussed, checked, and revised by a review committee. The committee was able to reach a consensus on all discrepancies found, thus ensuring idiomatic, semantic, conceptual, and experimental equivalence between the translated Arabic ISYQOL and the original English ISYQOL. In the Arabic translated version, we ensured the use of words and phrases that are simpler and easier to understand, as the patients were in the younger spectrum of age. In addition, as both the Arabic and English languages carry behind them a cultural background, some questions were modified to fit the culture of the Arabic-speaking population.

In ISYQOL question 1 “Are you afraid that your back problem may get worse?,” it was translated in Arabic to “evolve” rather than “get worse,” which was expected to be confusing in Arabic, especially for the younger patients. In ISYQOL question 2 “Are you worried about having back pain as an adult because of your back problem?,” it was translated in Arabic to “maturity” rather than “adulthood,” which are 2 different periods of life and have different meanings. In ISYQOL question 8 “Does the appearance of your back make you feel uncomfortable?” It was translated with the idea that “the patient being uncomfortable because other people are looking at his/her back” rather than the idea that “the patient being uncomfortable because of the appearance of his/her back.” Discrepancies were resolved by a committee review for these 3 questions (1, 2, and 8).

According to the study participants, they found that the Arabic version of ISYQOL questionnaire was more understandable and easier to fill when compared with Arabic version SRS-22. questionnaire. This might be due to the fact that SRS-22 questionnaire options are not identical in every question as in the ISYQOL. For example, in SRS-22 question 11 “Which one of the following best describes your medication usage for your back?”a participant had difficulty answering as he did not know what class his medication was under.

The Arabic version of the ISYQOL questionnaire had satisfactory reliability and validity when testing Arabic language-speaking patients with spinal deformities. The present study showed good test–retest reliability and internal consistency, measuring 0.798 using Cronbach's alpha for spine health, which is slightly better in value than for the different domains of the SRS-22r's Arabic version. Bracing for scoliosis and curve severity leads to a reduction in the quality-of-life score, whereas participation in sports improves it.^[30]^ In the present study, the internal consistency and reliability of the brace section of the Arabic version of the ISYQOL was acceptable, which might be attributed to the small number of patients wearing the brace in this cohort. Further analysis of this subset of patients’ cohorts in a large study with a larger sample may be required.

The results of the current study revealed a strong and significant correlation with the validated SRS-22r's Arabic version with a Pearson's correlation coefficient of 0.708 (*P* < .001), which is consistent with other published studies. However, the ISYQOL questionnaire is a more disease-specific questionnaire, as it allows direct HRQOL assessment and comparison between patients managed with brace prescription and patients who were not. In addition, the same comparison could be applied to the same patient prior to and after wearing a brace.^[30]^

Caronni showed that the ISYQOL is a valid tool for measuring HRQOL in young people with spinal deformities. It revealed that HRQOL was higher in adolescents than teenagers, in patients not attiring a brace than individuals who do, in people with better trunk appearance than in those with a worse trunk appearance, milder curves than severe ones, and in kyphosis against scoliosis patients, similar to the SRS-22r questionnaire, which is consistent with previous reports.^[37–40]^ However, ISYQOL was able to discriminate gender-related differences in quality of life, discriminate people having an enjoyable life quality, and portray the quality of life with finer details and sharper contrast compared to the SRS-22r questionnaire.^[30]^ Because the ISYQOL questionnaire is novel and was only tested on the Italian population, we feel that this study is important as the questionnaire is being tested on a different patient population and compared with the validated SRS-22r's Arabic version. Our findings support the results of Caronni et al. We were able to show that ISYQOL is a valid and useful tool in patients who have undergone surgical correction for spinal deformity and in patients with severe curves, despite not having questions directly investigating problems related to surgery or pain. This was done by the authors who developed the questionnaire because, in the framework of the Rasch analysis, a latent variable can be assessed and marked by multiple items, and 2 different items can mark the same variable, which in turn improves the ceiling effect. Some of the limitations of the current study are the relatively small sample size and the fact that the study was conducted in 1 region of Saudi Arabia; all study participants spoke Arabic with the same accent. Future studies with larger sample sizes and with participants from different Arab countries with different backgrounds are needed to test for differences in vocabulary used in different Arab countries and regions.

## 5 Conclusion

The Arabic version of the ISYQOL questionnaire has all the properties needed for health-related quality of life evaluation in young patients with spinal deformities. It can provide information about patients’ overall performance, health, and well-being. In addition, it provides valuable information about disease management in addition to radiological evaluation.

## Acknowledgments

The authors would like to acknowledge Dr Hani Alnajjar, Dr Sumayah Bafana, and Dr Abdulrahman Almatrafi for their help with the forward and backward translation. The acknowledgment and appreciation are also extended to the Faculty of Medicine of Umm Al-Qura University and the Department of Bioresearch for their contribution and help with the ethical approval and statistical analysis of this work.

## Author contributions

**Conceptualization:** Salah Fallatah, Shaker Emam, Ghamid Al-Ghamdi, Faisal Almatrafi.

**Data curation:** Salah Fallatah, Shaker Emam, Ghamid Al-Ghamdi, Faisal Almatrafi.

**Formal analysis:** Salah Fallatah, Shaker Emam, Ghamid Al-Ghamdi, Faisal Almatrafi.

**Investigation:** Salah Fallatah, Shaker Emam, Ghamid Al-Ghamdi.

**Methodology:** Salah Fallatah, Shaker Emam, Ghamid Al-Ghamdi, Faisal Almatrafi.

**Project administration:** Salah Fallatah, Shaker Emam, Ghamid Al-Ghamdi, Faisal Almatrafi.

**Resources:** Salah Fallatah, Shaker Emam, Ghamid Al-Ghamdi, Faisal Almatrafi.

**Software:** Salah Fallatah, Shaker Emam, Faisal Almatrafi.

**Supervision:** Salah Fallatah.

**Validation:** Salah Fallatah, Shaker Emam, Ghamid Al-Ghamdi, Faisal Almatrafi.

**Visualization:** Salah Fallatah, Shaker Emam, Faisal Almatrafi.

**Writing – original draft:** Salah Fallatah, Shaker Emam, Ghamid Al-Ghamdi, Faisal Almatrafi.

**Writing – review & editing:** Salah Fallatah, Shaker Emam, Ghamid Al-Ghamdi, Faisal Almatrafi.

## References

[1] Kelly MP Lurie JD Yanik EL. Operative versus nonoperative treatment for adult symptomatic lumbar scoliosis. J Bone Joint Surg Am 2019;101:338–52. 30801373 10.2106/JBJS.18.00483 PMC6738555

[2] Yagci G Ayhan C Yakut Y. Effectiveness of basic body awareness therapy in adolescents with idiopathic scoliosis: a randomized controlled study. J Back Musculoskelet Rehabil 2018;31:693–701. 29630516 10.3233/BMR-170868

[3] Monticone M Ambrosini E Cazzaniga D. Adults with idiopathic scoliosis improve disability after motor and cognitive rehabilitation: results of a randomised controlled trial. Eur Spine J 2016;25:3120–9. 27015689 10.1007/s00586-016-4528-y

[4] Negrini S Minozzi S Bettany-Saltikov J. Braces for idiopathic scoliosis in adolescents. Spine J 2016;41:1813–25. 10.1097/BRS.0000000000001887 27584672

[5] Han J Xu Q Yang Y Yao Z Zhang C. Evaluation of quality of life and risk factors affecting quality of life in adolescent idiopathic scoliosis. Intractable Rare Dis Res 2015;4:12–6. 25674383 10.5582/irdr.2014.01032 PMC4322590

[6] Miekisiak G Kollataj M Dobrogowski J. Cross-cultural adaptation and validation of the Polish version of the Core Outcome Measures Index for low back pain. Eur Spine J 2013;22:995–1001. 23229802 10.1007/s00586-012-2607-2 PMC3657047

[7] Haher TR Gorup JM Shin TM. Results of the scoliosis research society instrument for evaluation of surgical outcome in adolescent idiopathic scoliosis: a multicenter study of 244 patients. Spine J 1999;24:1435–40. 10.1097/00007632-199907150-00008 10423788

[8] Teles AR Khoshhal KI Falavigna A. Why and how should we measure outcomes in spine surgery? J Taibah Univ Med Sci 2016;11:91–7.

[9] Algarni FS Alotaibi AN Altowaijri AM Al-Sobayel H. Cross-cultural adaptation and validation of the Arabic version of musculoskeletal health questionnaire (Msk-hq-ar). Int J Environ Res Public Health 2020;17:01–11. 10.3390/ijerph17145168 PMC7400235 32709115

[10] Meng ZD Li TP Xie XH Luo C Lian XY Wang ZY. Quality of life in adolescent patients with idiopathic scoliosis after brace treatment. Med (United States) 2017;96:01–9. 10.1097/MD.0000000000006828 PMC5428595 28489761

[11] Storheim K Brox JI Løchting I Werner EL Grotle M. Cross-cultural adaptation and validation of the Norwegian version of the Core Outcome Measures Index for low back pain. Eur Spine J 2012;21:2539–49. 22695701 10.1007/s00586-012-2393-x PMC3508207

[12] Caronni A Sciumè L Donzelli S Zaina F Negrini S. ISYQOL: a Rasch-consistent questionnaire for measuring health-related quality of life in adolescents with spinal deformities. Spine J 2017;17:1364–72. 28529002 10.1016/j.spinee.2017.05.022

[13] Doi T Inoue H Arai Y. Reliability and validity of a novel quality of life questionnaire for female patients with adolescent idiopathic scoliosis: Scoliosis Japanese Questionnaire-27: a multicenter, cross-sectional study. BMC Musculoskelet Disord 2018;19:01–10. 10.1186/s12891-018-2025-7 PMC5883312 29615021

[14] Schlösser TPC Stadhouder A Schimmel JJP Lehr AM Van Der Heijden GJMG Castelein RM. Reliability and validity of the adapted Dutch version of the revised Scoliosis Research Society 22-item questionnaire. Spine J 2014;14:1663–72. 24360746 10.1016/j.spinee.2013.09.046

[15] Caronni A Zaina F Negrini S. Improving the measurement of health-related quality of life in adolescent with idiopathic scoliosis: The SRS-7, a Rasch-developed short form of the SRS-22 questionnaire. Res Dev Disabil 2014;35:784–99. 24521663 10.1016/j.ridd.2014.01.020

[16] Alamrani S Rushton A Gardner A Falla D Heneghan NR. Outcome measures evaluating physical functioning and their measurement properties in adolescent idiopathic scoliosis: a protocol for a systematic review. BMJ Open 2020;10:01–7. 10.1136/bmjopen-2019-034286 PMC7170637 32241788

[17] Haidar RK Kassak K Masrouha K Ibrahim K Mhaidli H. Reliability and validity of an adapted Arabic version of the scoliosis research society-22r questionnaire. Spine (Phila Pa 1976) 2015;40:E971–7. 25929208 10.1097/BRS.0000000000000956

[18] Beauséjour M Joncas J Goulet L. Reliability and validity of adapted french canadian version of scoliosis research society outcomes questionnaire (SRS-22) in Quebec. Spine (Phila Pa 1976) 2009;34:623–8. 19282743 10.1097/BRS.0b013e3181973e58

[19] Hashimoto H Sase T Arai Y Maruyama T Isobe K Shouno Y. Validation of a Japanese version of the Scoliosis Research Society-22 patient questionnaire among idiopathic scoliosis patients in Japan. Spine (Phila Pa 1976) 2007;32:141–6. 10.1097/01.brs.0000255220.47077.33 17304124

[20] Mousavi SJ Mobini B Mehdian H. Reliability and validity of the Persian Version of the Scoliosis Research Society-22r Questionnaire. Spine J 2010;35:784–9. 10.1097/BRS.0b013e3181bad0e8 20228713

[21] Glowacki M Misterska E Laurentowska M Mankowski P. Polish adaptation of scoliosis research society-22 questionnaire. Spine (Phila Pa 1976) 2009;34:1060–5. 19404181 10.1097/BRS.0b013e31819c1ec3

[22] Carriço G Meves R Avanzi O. Cross-cultural adaptation and validity of an adapted Brazilian Portuguese version of scoliosis research society–30 questionnaire. Spine (Phila Pa 1976) 2012;37:E60–3. 22045004 10.1097/BRS.0b013e31823c7cd6

[23] Bago J Climent JM Ey A Perez-Grueso FJS Izquierdo E. The Spanish Version of the SRS-22 patient questionnaire for idiopathic scoliosis: transcultural adaptation and reliability analysis. Spine (Phila Pa 1976) 2004;29:1676–80. 15284516 10.1097/01.brs.0000132306.53942.10

[24] Lee JS Lee DH Suh KT Kim JI Lim JM Goh TS. Validation of the Korean version of the Scoliosis Research Society-22 questionnaire. Eur Spine J 2011;20:1751–6. 21670944 10.1007/s00586-011-1872-9 PMC3175885

[25] Danielsson AJ Romberg K. Reliability and validity of the Swedish Version of the Scoliosis Research Society–22 (SRS-22r) patient questionnaire for idiopathic scoliosis. Spine (Phila Pa 1976) 2013;38:1875–84. 23846501 10.1097/BRS.0b013e3182a211c0

[26] Alanay A Cil A Berk H. Reliability and validity of adapted Turkish version of scoliosis research society-22 (SRS-22) Questionnaire. Spine (Phila Pa 1976) 2005;30:2464–8. 16261127 10.1097/01.brs.0000184366.71761.84

[27] Climent JM Bago J Ey A Perez-Grueso FJS Izquierdo E. Validity of the Spanish Version of the Scoliosis Research Society-22 (SRS-22) Patient Questionnaire. Spine (Phila Pa 1976) 2005;30:705–9. 15770189 10.1097/01.brs.0000155408.76606.8f

[28] Zhao L Zhang Y Sun X Du Q Shang L. The Scoliosis Research Society-22 questionnaire adapted for adolescent idiopathic scoliosis patients in China: reliability and validity analysis. J Child Orthop 2007;1:351–5. 19308531 10.1007/s11832-007-0061-1 PMC2656752

[29] Dabaghi S Esmaielzadeh F Rohani C. Application of rasch analysis for development and psychometric properties of adolescents’ quality of life instruments: a systematic review. Adolesc Health Med Ther 2020;11:173–97. 33204203 10.2147/AHMT.S265413 PMC7666979

[30] Caronni A Donzelli S Zaina F Negrini S. The Italian Spine Youth Quality of Life questionnaire measures health-related quality of life of adolescents with spinal deformities better than the reference standard, the Scoliosis Research Society 22 questionnaire. Clin Rehabil 2019;33:1404–15. 30977381 10.1177/0269215519842246

[31] Beauford JE Nagashima Y Wu M-H. Using translated instruments in research. J Coll Teach Learn 2011;6:77–82.

[32] Sperber AD. Translation and validation of study instruments for cross-cultural research. Gastroenterology 2004;126:124–8. 10.1053/j.gastro.2003.10.016 14978648

[33] Tsang S Royse CF Terkawi AS. Guidelines for developing, translating, and validating a questionnaire in perioperative and pain medicine. Saudi J Anaesth 2017;11:S80–9. 28616007 10.4103/sja.SJA_203_17 PMC5463570

[34] Koller M Kantzer V Mear I. The process of reconciliation: evaluation of guidelines for translating quality-of-life questionnaires. Expert Rev Pharmacoecon Outcomes Res 2012;12:189–97. 22458620 10.1586/erp.11.102

[35] Lenke LG Betz RR Harms J. Adolescent idiopathic scoliosis. A new classification to determine extent of spinal arthrodesis. J Bone Jt Surg 2001;83:1169–81. 11507125

[36] Baba MR Shenoy RM Soman A. Cross-cultural adaption and validity of an adapted Kannada (South Indian language) version of Scoliosis Research Society (SRS-30) Questionnaire for idiopathic scoliosis. Spine Deform 2021;9:327–31. 33400229 10.1007/s43390-020-00242-x

[37] Thompson JY Williamson EM Williams MA Heine PJ Lamb SE. Effectiveness of scoliosis-specific exercises for adolescent idiopathic scoliosis compared with other non-surgical interventions: a systematic review and meta-analysis. Physiother 2019;105:214–34. 10.1016/j.physio.2018.10.004 30824243

[38] Schwieger T Campo S Weinstein SL Dolan LA Ashida S Steuber KR. Body image and quality-of-life in untreated versus brace-treated females with adolescent idiopathic scoliosis. Spine (Phila Pa 1976) 2016;41:311–9. 26555827 10.1097/BRS.0000000000001210 PMC4736292

[39] Mariconda M Andolfi C Cerbasi S Servodidio V. Effect of surgical correction of adolescent idiopathic scoliosis on the quality of life: a prospective study with a minimum 5-year follow-up. Eur Spine J 2016;25:3331–40. 26984879 10.1007/s00586-016-4510-8

[40] Tones M Moss N Polly DW. A review of quality of life and psychosocial issues in scoliosis. Spine (Phila Pa 1976) 2006;31:3027–38. 17173000 10.1097/01.brs.0000249555.87601.fc

